# Integration of Fine Model-Based Decomposition and Guard Filter for Ship Detection in PolSAR Images

**DOI:** 10.3390/s21134295

**Published:** 2021-06-23

**Authors:** Dongsheng Liu, Ling Han

**Affiliations:** 1 School of Geology Engineering and Geomatics, Chang’an University, Xi’an 710054, China; 2018026016@chd.edu.cn; 2 PIESAT Information Technology Co., Ltd., Beijing 100195, China; 3 Key Laboratory of Land Consolidation in Shaanxi Province, Xi’an 710054, China; 4 School of Land Engineering, Chang’an University, Xi’an 710054, China

**Keywords:** ship detection, polarimetric synthetic aperture radar (PolSAR), fine eight-component decomposition, guard filter

## Abstract

Ship detection with polarimetric synthetic aperture radar (PolSAR) has gained extensive attention due to its widespread application in maritime surveillance. Nevertheless, designing identifiable features to realize accurate ship detection is still challenging. For this purpose, a fine eight-component model-based decomposition scheme is first presented by incorporating four advanced physical scattering models, thus accurately describing the dominant and local structure scattering of ships. Through analyzing the exclusive scattering mechanisms of ships, a discriminative ship detection feature is then constructed from the derived contributions of eight kinds of scattering components. Combined with a spatial information-based guard filter, the efficacy of the feature is further amplified and thus a ship detector is proposed which fulfills the final ship detection. Several qualitative and quantitative experiments are conducted on real PolSAR data and the results demonstrate that the proposed method reaches the highest figure-of-merit (FoM) factor of 0.96, which outperforms the comparative methods in ship detection.

## 1. Introduction

Since polarimetric synthetic aperture radar (PolSAR) can offer intuitive physical explanations for scattering behaviors that could make targets or structures identifiable, ship detection with PolSAR images has received continuous attention and is crucial for maritime surveillance [1,2,3].

Based on the statistical analysis, the constant false alarm rate (CFAR) is one of the popularly used techniques for ship detection [4,5]. In the case of unknown prior information, the CFAR enjoys favorable detection performance since ships have more intense scattering responses compared with sea clutters. Nevertheless, influenced by the diverse radar platform parameter and complex sea condition, the accurate statistical modeling of sea clutter and the corresponding parameter estimation are generally arduous and complicated. As artificial intelligence develops, an emerging method is to train a deep neural network to distinguish the important features. In this vein, the convolutional neural network (CNN) based category is popular as it can approximate the underlying function which describes the backscatter characteristics of ships and automatically extract low- and high-dimensional image structural features [6]. For instance, Zhang et al. [7] designed a depthwise separable CNN to realize ship detection. Jin et al. [8] proposed a patch to pixel CNN to enhance the small ship detection performance. However, the application of deep learning is limited by the massive sample training and the interpretability is still suspicious.

In contrast, characterizing scattering behaviors and extracting appropriate features are more promising and practical. Starting from the exploitation of intensity information, Novak et al. [9] and De Graff [10], respectively, proposed the polarimetric whitening filter (PWF), the total power (SPAN), and the power maximization synthesis (PMS) detectors for ship discrimination. Using the intrinsic different symmetry of sea clutter and manmade targets, Velotto et al. [11] designed the reflection symmetry (RS) to detect ships. Through analyzing the scattering mechanism, Sugimoto et al. [12] adopted the four-component polarimetric decomposition as a band-stop detector (Pt-Ps) to hinder the scattering contribution from the sea clutter. Zhang et al. [13] constructed a covariance difference matrix and then applied the four-component decomposition to it for ship detection (DBSPc). Further, Wei et al. [14] proposed a ship detection method based on cross-correlation between the volume and helix scattering mechanisms (VC-CR). In addition, Marino et al. [15] designed the polarimetric notch filter (PNF) using the polarimetric complex scattering vectors to discriminate ships from the clutter.

Although these methods have achieved, with various levels of success, ship detection, certain deficiencies still exist. First, the intensity-based detectors are susceptible to target-to-clutter ratio (TCR) and may endure severe performance degradation in the case of low TCRs. Second, although the clutter interference can be suppressed with the scattering mechanism features, the scattering model may lose its effectiveness when dealing with complex structures and backgrounds. Finally, yet importantly, detection of ships with small dimensions and weak backscattering in rough sea state scenarios is always troublesome.

To deal with these issues, this paper proposes a PolSAR image ship detection method through integrating the fine model-based decomposition (MBD) and guard filter. The MBD is considered because it allows the discrimination of scattering behaviors in polarization signatures by associating them with physical meanings [16,17,18]. This work contains the following aspects; first, to accurately describe the local structure scattering of ships, four advanced scattering models are introduced and therefore an eight-component decomposition is put forward; second, by analyzing the scattering mechanism differences between sea clutter and ships, a novel detection feature is designed based on the derived scattering contributions; third, considering the utilization of spatial information, a guard filter is constructed and incorporated to further amplify the efficacy of the feature; finally, experiments are conducted on real PolSAR data to qualitatively and quantitatively assess the proposed method with comparative methods and the results demonstrate that the proposed method enjoys optimal detection performance and can effectively enhance the TCR. The main contributions introduced by this paper lie in: (1) The proposition of eight-component model-based decomposition, which characterizes the ship scattering according to its local complex structures; (2) The construction of scattering contribution-based detection feature, which highlights the scattering differences between the ships and sea clutters; (3) The design of a guard filter-based detector, which excludes the interferences and amplifies the efficacy of the detection feature.

## 2. Methodology

### 2.1. Advanced Physical Scattering Models

As is generally known, the most significant structure of a ship is dihedral which can induce an intense double-bounce co-polarized response. However, once the dihedral orientation deviates from the radar flight direction, remarkable cross-polarized power instead of co-polarized power will be generated [19,20,21]. In this case, traditional MBD methods generally suffer deficiencies in accurate characterization because they cannot recognize this change for the improper scattering modeling. In existing studies, since the cross scattering model (CSM) [22] can adaptively integrate the orientation information into the dihedral scattering modeling, it is a preferable option in scattering understanding of the rapid transition between co-polarized and cross-polarized powers. Considering this, the eight-component polarimetric decomposition scheme adopts the CSM (i.e., the first advanced physical scattering model) to deal with this problem. To be specific, the CSM ${\left[T\right]}_{\mathrm{CRO}}$ is derived by integrating the dominant polarimetric orientation (PO) angle information into the cosine distribution to perform ensemble averaging of dihedrals. The cosine squared distribution is assumed because it is generally used for vertical structures whereas a uniform distribution is suitable for randomly distributed dipoles [22].

(1) [T]CRO=[000015−cos(4θOA)3000015+cos(4θOA)30]withθOA=14(tan−12Im(T23)T22−T33)

Thereinto, $\theta_{\mathrm{OA}}$ represents the PO angle and the $T_{22}$, $T_{23}$, and $T_{33}$ terms represent the coherency matrix elements, which are presented in Equation (5). It has been proven that the CSM can effectively separate the cross-polarized powers of rotated dihedrals from the overall cross-polarized powers. Therefore, the incorporation of the CSM is expected to retain the cross-polarized components caused by rotated dihedrals which are mistakenly assigned to other structures.

On the other hand, the coherency matrix of a ship pixel generally does not subject to the reflection symmetry assumption due to the heterogeneous scattering and strong depolarization effect [23]. In this case, the real and imaginary parts of $T_{13}$ and $T_{23}$ terms are non-zero. Nevertheless, most of the MBD methods do not consider the modeling of implicit scattering related to these terms and they generally force the $T_{13}$ and $T_{23}$ terms to be zero instead. Accordingly, important polarimetric information is lost and the practicability is limited to a great extent. In order to adequately utilize the matrix information and to break the assumption of reflection symmetry, certain scattering models should be involved to account for the $T_{13}$ and $T_{23}$ terms. To address this, three extra advanced physical scattering models, namely the ±45° oriented dipole (OD), ±45° oriented quarter-wave (OQW) and mixed dipole (MD) scattering models [24,25] are ulteriorly incorporated into the eight-component decomposition scheme.

Referring to [24,25], the ±45° OD, ±45° OQW, and MD scattering model are acquired from the spatial combination of dipoles with different orientations. Thereinto, the ±45° OD scattering model is modeled by a dipole oriented at 45° (−45°) about the radar line of sight. The ±45° OQW scattering is regarded as the summation of scattering of the ±45° oriented dipoles locating at different distances with d=0, d=λ/8, d=3λ/8. The MD scattering is assumed to occur on a particular configuration of dipoles, i.e., a horizontal dipole locates at d=0 and a 45° oriented dipole locates at d=λ/4. Thus, the coherency matrices can be derived through the inner product of scattering vectors in Pauli basis:

(2) $${\left[T\right]}_{\mathrm{OD}}^{\pm 45^{{}^{\circ}}}=\mathrm{vec}({\left[S\right]}_{\mathrm{OD}}^{\pm 45^{{}^{\circ}}})\cdot \mathrm{vec}{\left({\left[S\right]}_{\mathrm{OD}}^{\pm 45^{{}^{\circ}}}\right)}^{H}=\frac{1}{2}\left[\begin{array}{ccc} 1 & 0 & \pm 1\\ 0 & 0 & 0\\ \pm 1 & 0 & 1 \end{array}\right].$$

(3) $${\left[T\right]}_{\mathrm{OQW}}^{\pm 45^{{}^{\circ}}}=\mathrm{vec}({\left[S\right]}_{\mathrm{OQW}}^{\pm 45^{{}^{\circ}}})\cdot \mathrm{vec}{\left({\left[S\right]}_{\mathrm{OQW}}^{\pm 45^{{}^{\circ}}}\right)}^{H}=\frac{1}{2}\left[\begin{array}{ccc} 1 & 0 & \mp j\\ 0 & 0 & 0\\ \pm j & 0 & 1 \end{array}\right].$$

(4) $${\left[T\right]}_{\mathrm{MD}}=\mathrm{vec}({\left[S\right]}_{\mathrm{MD}})\cdot \mathrm{vec}{\left({\left[S\right]}_{\mathrm{MD}}\right)}^{H}=\frac{1}{2}\left[\begin{array}{ccc} 0 & 0 & 0\\ 0 & 1 & \pm 1\\ 0 & \pm 1 & 1 \end{array}\right].$$

thereinto, j represents the imaginary unit. The notations vec(⋅) and H represent the operations of matrix vectorization and conjugate transpose, respectively.

Here, [S] represents Sinclair matrix and it is acquired by the linear combination of Sinclair matrices of dipoles with certain orientations and spatial locations. These three combinations are in accordance with the actual physical meaning since the $T_{13}$/$T_{23}$ term can be considered as a coupling between the first/second and third scattering vectors on a Pauli basis. In actual ship scattering, these configurations are existing and reasonable because the complex superstructures composed of dipole-like structures (such as towers, antennas, and guardrails) can induce distinct ±45° OD, ±45° OQW, and MD scattering. The introduction of scattering mechanisms of these local structures is helpful for comprehensively understanding and characterizing the ship scatterings. Notice that these three scattering models correspond to the real part of $T_{13}$, the imaginary part of $T_{13}$, and the real part of $T_{23}$. The imaginary part of $T_{23}$ does not reflect because the helix scattering in Yamaguchi four-component decomposition [26] has already accounted for it.

### 2.2. Eight-Component Model-Based Decomposition

Under the circumstance of monostatic backscattering, the Sinclair matrix subjects to the reciprocity condition and thus leading to the following coherency matrix:

(5) $$\langle \left[T\right]\rangle =\langle k_{3p}k_{3p}^{H}\rangle =\left[\begin{array}{ccc} T_{11} & T_{12} & T_{13}\\ T_{21} & T_{22} & T_{23}\\ T_{31} & T_{32} & T_{33} \end{array}\right]$$

where the notations $k_{3p}$ and 〈⋅〉 denote the Pauli vector and the spatial averaging operations, respectively. Combing the aforementioned advanced physical scattering models, the eight-component decomposition is proposed as the weighted sum of eight kinds of scattering mechanisms:

(6) $$\begin{array}{ll} \langle \left[T\right]\rangle & =f_{S}{\left[T\right]}_{S}+f_{D}{\left[T\right]}_{D}+f_{H}{\left[T\right]}_{H}+f_{V}{\left[T\right]}_{V}\\ & +f_{\mathrm{CRO}}{\left[T\right]}_{\mathrm{CRO}}+f_{\mathrm{OD}}{\left[T\right]}_{\mathrm{OD}}^{\pm 45^{{}^{\circ}}}+f_{\mathrm{OQW}}{\left[T\right]}_{\mathrm{OQW}}^{\pm 45^{{}^{\circ}}}+f_{\mathrm{MD}}{\left[T\right]}_{\mathrm{MD}} \end{array}$$

The mathematical expressions of ${\left[T\right]}_{S}$, ${\left[T\right]}_{D}$, ${\left[T\right]}_{H}$ and ${\left[T\right]}_{V}$ are given in Equation (7) [26,27] and they correspond to surface, double-bounce, helix, and volume scattering structures, respectively. Thereinto, α and β represent the undetermined model parameters of double-bounce and surface scattering, respectively. Here, $f_{S}$, $f_{D}$, $f_{H}$, $f_{V}$, $f_{\mathrm{CRO}}$, $f_{\mathrm{OD}}$, $f_{\mathrm{OQW}}$, and $f_{\mathrm{MD}}$ denote the corresponding weights to be computed:

(7) $$\begin{array}{l} {\left[T\right]}_{S}=\left[\begin{array}{ccc} 1 & \beta^{*} & 0\\ \beta & |\beta {|}^{2} & 0\\ 0 & 0 & 0 \end{array}\right],{\left[T\right]}_{D}=\left[\begin{array}{ccc} |\alpha {|}^{2} & \alpha & 0\\ \alpha^{*} & 1 & 0\\ 0 & 0 & 0 \end{array}\right]\\ {\left[T\right]}_{H}=\frac{1}{2}\left[\begin{array}{ccc} 0 & 0 & 0\\ 0 & 1 & \pm j\\ 0 & \mp j & 1 \end{array}\right],{\left[T\right]}_{V}=\frac{1}{4}\left[\begin{array}{ccc} 2 & 0 & 0\\ 0 & 1 & 0\\ 0 & 0 & 1 \end{array}\right]. \end{array}$$

Based on Equation (6), we can obtain several equation sets, as given in Equation (8). From Equation (8), it is obvious that there exist ten unknowns and nine observations. To deal with the underdetermined issue, one of the unknowns should be reduced by making certain assumptions. Similar to [28], the relative value of $T_{11}$ and $T_{22}$ in the remaining matrix (after subtracting the helix, ±45° OD, ±45° OQW, and MD scattering components from the original matrix), namely $T_{11}-T_{22}+f_{H}/2-f_{\mathrm{OD}}/2-f_{\mathrm{OQW}}/2+f_{\mathrm{MD}}/2$ is used. If $T_{11}-T_{22}+f_{H}/2-f_{\mathrm{OD}}/2-f_{\mathrm{OQW}}/2+f_{\mathrm{MD}}/2>0$, then the residual is judged as having surface scattering as dominant, thus $f_{D}=0$. Otherwise, the dominant scattering in the residual is double-bounce scattering, leading to $f_{S}=0$. However, obtaining the analytic solution is still complicated even though the resulting equations are compact. Given this, the subtraction of the equations regarding the $T_{22}$ and $T_{33}$ terms in Equation (8) is implemented and thus acquiring the simplified expressions in Equation (9):

(8) fS+fD|α|2+fV2+fOD2+fOQW2=T11fS|β|2+fD+fV4+fH2+fMD2+fCRO15−cos(4θOA)30=T22fV4+fH2+fCRO15+cos(4θOA)30+fOD2+fOQW2+fMD2=T33fSβ∗+fDα=T12fH2=|Im(T23)|,fOD2=|Re(T13)|fOQW2=|Im(T13)|,fMD2=|Re(T23)|.

(9) $$\begin{array}{l} T_{11}-T_{22}+\frac{f_{H}}{2}-\frac{f_{\mathrm{OD}}}{2}-\frac{f_{\mathrm{OQW}}}{2}+\frac{f_{\mathrm{MD}}}{2}>0:\\ f_{S}|\beta {|}^{2}-f_{\mathrm{CRO}}\frac{\cos(4\theta_{\mathrm{OA}})}{15}-\frac{f_{\mathrm{OD}}}{2}-\frac{f_{\mathrm{OQW}}}{2}=T_{22}-T_{33}\\ T_{11}-T_{22}+\frac{f_{H}}{2}-\frac{f_{\mathrm{OD}}}{2}-\frac{f_{\mathrm{OQW}}}{2}+\frac{f_{\mathrm{MD}}}{2}<0:\\ f_{D}-f_{\mathrm{CRO}}\frac{\cos(4\theta_{\mathrm{OA}})}{15}-\frac{f_{\mathrm{OD}}}{2}-\frac{f_{\mathrm{OQW}}}{2}=T_{22}-T_{33}. \end{array}$$

Further, the $f_{\mathrm{CRO}}\cdot \cos(4\theta_{\mathrm{OA}})/15$ term can be omitted since $f_{\mathrm{CRO}}$ and $\cos(4\theta_{\mathrm{OA}})/15$ are both small relative to other terms. On this occasion, $f_{S}$ and $f_{D}$ can be directly solved from the $T_{12}$ term. In case of the weight of surface or double-bounce scattering is ascertained, the rest of the weights can be determined, as given in Equations (10) and (11):

(10) T11−T22+fH2−fOD2−fOQW2+fMD2>0:fD=0,fH=2|Im(T23)|,fOD=2|Re(T13)|,fOQW=2|Im(T13)|fMD=2|Im(T23)|,fS=|T12|2T11−T22+fOD2+fOQW2fV=2(T11−fS−fOD2−fOQW2),fCRO=4T33−2fH−fV−2fOD−2fOQW−2fMD30+2cos(4θOA)15.

or

(11) T11−T22+fH2−fOD2−fOQW2+fMD2<0:fS=0,fH=2|Im(T23)|,fOD=2|Re(T13)|,fOQW=2|Im(T13)|fMD=2|Im(T23)|,fD=T11−T22+fOD2+fOQW2fV=2(T11−fOD2−fOQW2−|T12|2fD),fCRO=4T33−2fH−fV−2fOD−2fOQW−2fMD30+2cos(4θOA)15.

Finally, the contributions of the surface, double-bounce, volume, helix, cross, ±45° OD, ±45° OQW, MD scattering are respectively calculated as:

(12) $$\begin{array}{l} P_{S}=f_{S}(1+|\beta {|}^{2}),P_{D}=f_{D}(1+|\alpha {|}^{2}),P_{V}=f_{V},P_{H}=f_{H},\\ P_{\mathrm{CRO}}=f_{\mathrm{CRO}},P_{\mathrm{OD}}=f_{\mathrm{OD}},P_{\mathrm{OQW}}=f_{\mathrm{OQW}},P_{\mathrm{MD}}=f_{\mathrm{MD}}. \end{array}$$

### 2.3. Detection Feature Construction

As is generally known, for the sea clutter, the surface scattering is the primary contribution and absolutely dominates in the total backscattering. Meanwhile, the dominant scattering mechanism of the ship interpreted by the fine eight-component decomposition is mainly double-bounce scattering. This is acknowledged because ships mainly consist of dihedral structures, such as broadside-sea configurations and deck hatches. Due to the existence of dihedral structures, the orientation shifts will also induce obvious cross scattering. To facilitate understanding, double-bounce and cross scattering are collectively called dihedral scattering. Therefore, to highlight the scattering difference, the ratio of dihedral and surface scattering contributions can be regarded as a candidate feature for detection.

On the other hand, since sea clutter belongs to naturally distributed targets, the reflection symmetry is generally present, i.e., $\langle \left|S_{\mathrm{HH}}S_{\mathrm{HV}}^{*}\right|\rangle =\langle \left|S_{\mathrm{HV}}S_{\mathrm{VV}}^{*}\right|\rangle =0$ [29]. In this case, the helix, ±45° OD, ±45° OQW, and MD scattering (collectively called compound scattering) contributions which calculated from the $T_{13}$ and $T_{23}$ terms can be neglected. In comparison, since ships are composed of complex superstructures, considerable compound scattering are exhibited generally. Accordingly, the detection feature is finally proposed as:

(13) $$Fea_{\mathrm{Ship}}=\log\frac{P_{D}+P_{\mathrm{CRO}}+P_{\mathrm{COM}}}{P_{S}}=\log\frac{P_{D}+P_{\mathrm{CRO}}+P_{H}+P_{\mathrm{OD}}+P_{\mathrm{OQW}}+P_{\mathrm{MD}}}{P_{S}}$$

This feature is constructed with the following properties: A relatively large value of $Fea_{\mathrm{Ship}}$ can be observed for ships compared with sea clutter since the contribution of dihedral and compound scattering is weak for sea clutter while surface scattering is the dominant contribution. Intuitively, this feature can effectively enhance the TCR and improve detection performance.

### 2.4. Guard Filter-Based Ship Detector

Directly applying the constructed feature from pixel-to-pixel for ship detection may cause the occurrence of outliers due to the influence of noises as well as the impurity of target estimation. Additionally, enhancing the feature difference between ships and sea clutter as much as possible is crucial for small ship detection. Thus, how to skillfully exploit the spatial information for further improving the TCR is desirable. Considering this, this section designs a guard filter-based ship detector in order to amplify the feature efficacy through the window sliding processing.

**Figure 1:**
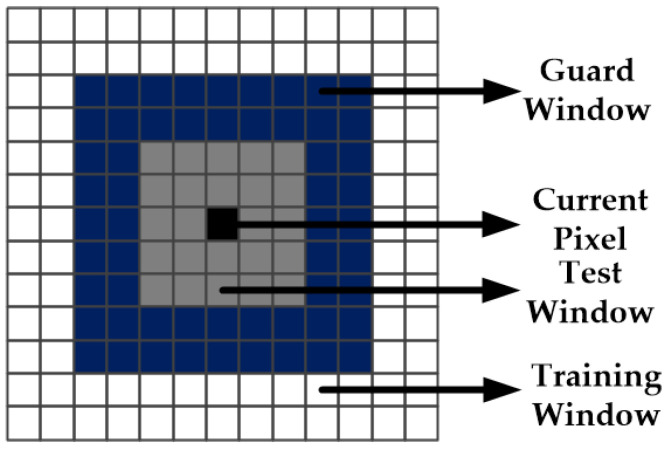
The configuration of guard filter.

As shown in Figure 1, the guard filter is composed of three levels of structures. The target cell is on the inside, which denotes a test window. The guard window is in the middle and the training window (the sea clutter) is on the outside. The guard window prevents ship pixels in the test window from interfering with sea clutter pixels while ship pixels can be excluded as outliers for sea clutter in the training window. Accordingly, the guard filter-based ship detector is finally presented as:

(14) $$\begin{array}{ll} Det_{\mathrm{Ship}} & =\log\frac{{\langle P_{D}+P_{\mathrm{CRO}}+P_{\mathrm{COM}}\rangle }_{\mathrm{Test}}}{{\langle P_{S}\rangle }_{\mathrm{Train}}}-\log\frac{{\langle P_{D}+P_{\mathrm{CRO}}+P_{\mathrm{COM}}\rangle }_{\mathrm{Train}}}{{\langle P_{S}\rangle }_{\mathrm{Train}}}\\ & =\log\frac{{\langle P_{D}+P_{\mathrm{CRO}}+P_{\mathrm{COM}}\rangle }_{\mathrm{Test}}}{{\langle P_{D}+P_{\mathrm{CRO}}+P_{\mathrm{COM}}\rangle }_{\mathrm{Train}}} \end{array}$$

where, the notations $<\cdot {>}_{\mathrm{Test}}$ and $<\cdot {>}_{\mathrm{Train}}$ denote the assemble average of the test and training windows, respectively. It can be seen that through the manipulation in Equation (14), the target and clutter are initially screened and the influence of outliers (noises) is reduced. Meanwhile, the sub-features $P_{D}+P_{\mathrm{CRO}}+P_{\mathrm{COM}}$ and $P_{S}$ are applied to the corresponding target/sea clutter pixels to derive the final ship detector $Det_{\mathrm{Ship}}$ such that the scattering difference is expected to be significantly amplified. As a result, the characteristics of the guard filter-based ship detector are summarized as follows:

(1) If the training window is located at a complete sea clutter region, the pixel scattering in the test window are homogenous, thus the scattering contributions in the test and training windows are approximately equal. As a result, $Det_{\mathrm{Ship}}$ is close to zero;

(2) If a ship pixel falls into the test window, the ship scattering contributions in the test window must exceed those in the guard and training window. In this being, $Det_{\mathrm{Ship}}$ will be positive and large, thus the ship target can be effectively outlined;

(3) If a ship target occupies both the guard and training window, the pixels in the test window almost all belong to the sea clutter category. The ship scattering contributions in the test window are smaller than those in the training window, therefore $Det_{\mathrm{Ship}}$ may be negative.

As a result, if the test window consists of ship pixels, $Det_{\mathrm{Ship}}$ will value at a relatively high level and, thus, a feature map with very strong TCR can be acquired to detect ship targets. As we know, the guard filter designed in many pieces of literature is used to prevent the target pixels from contaminating clutter estimation. However, when one comprehends the design of the guard filter in this study, it should be noted that the guard filter and detection feature perform and function jointly. On the one hand, by exploiting the spatial information, the guard filter not only reduces the influence of outliers (noises) that occur in the pixel-to-pixel application of the detection feature but also prevents the target pixels from contaminating clutter estimation. On the other hand, the existing guard filters are generally designed to perform sliding window operations on the feature image with an unchanged expression of detection feature (i.e., simply select the target and clutter pixels for feature utilization). Meanwhile in our work, through combing the detection feature and guard filter, the mathematical form of guard filter-based detector clearly differs from the one of detection feature. This, in fact, indicates that the detector has eliminated the effect of surface scattering contribution (the sea clutter), which can amplify the efficacy of the detection feature, thus enhancing the TCR.

Considering the demand of the approximate number of ship pixels and sea clutter pixels for the detector, and according to the ship size and data resolution, the size difference between the side lengths of the guard and training windows is set to be a constant (4 pixels for the entire window) in this work. Thus, only the sizes of test and guard windows need to be ascertained. Empirically, the size of the test window is set as 3 × 3 pixels. For the size of the guard window, it is set as 31 × 31 pixels through investigating its influence on detection performance in the following Discussion section. The above parameter settings are reasonable because, for small ships which require a larger TCR, a large training window along with a small test window is encouraged.

Finally, the detection is fulfilled by selecting the threshold according to the histogram thresholding method [30,31]. Since the proposed detector corresponds to the presence of ship scattering saliency, a histogram of the specific characteristic discriminative for the detection is available. The threshold selection procedure is briefly summarized as follows. First, a certain amount of ship pixels is selected as training samples. Subsequently, by means of the isolation effect of the guard window, the detector (feature) histograms of ships and clutters are estimated. At the last, the intersection point of the histograms, i.e., the bottom of the valley between two peaks on the histograms is selected as the detection threshold.

## 3. Experimental Results

### 3.1. Datasets

To carry out the assessment of the proposed detection method, the results reported here are tested on Gaofen-3 (GF3) C-band data acquired over the Pearl River Estuary, China, and AIRSAR L-band data acquired over Tokyo Bay, Japan. The GF3 data was acquired on 5 August 2017, which has a resolution with 8.00 m × 8.00 m in the ground area (azimuth × range). Meanwhile, the resolution of AIRSAR data corresponds to 5.00 m × 2.80 m in the ground area and it was acquired on 2 October 2000.

**Figure 2:**
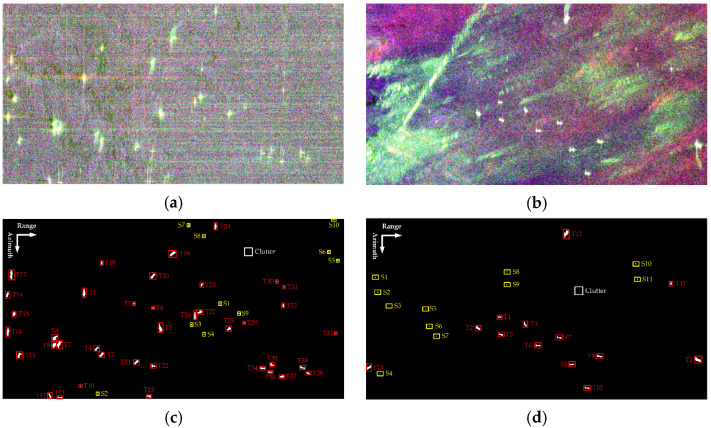
Pauli color-coding PolSAR data and their corresponding ground truths for different study sites. (**a**,**c**) GF3 data. (**b**,**d**) AIRSAR data. The strong and weak targets are marked by red and yellow rectangles, respectively.

Figure 2a,b displays the fully polarimetric GF3 and AIRSAR data with Pauli color-coding, where the red pixels are double-bounce events, green pixels are volume scatter events, and blue pixels are single bounce scatter events. The ground truth is defined according to a comprehensive interpretation with optical and polarimetric information. To be specific, we do not have the automatic identification system (AIS) information corresponding to the aforementioned data. Instead, we here determine the locations of ships mainly by visual inspection, together with referring to the published paper (see [13,32]) where the same data sets are used. Visual inspection is reasonable due to the fact that ships usually have stronger backscattering than the surrounding clutter and are displayed as bright spots in PolSAR images. There are 49 and 24 ships in Figure 2c,d), respectively. The large ships are outlined by red rectangles, while the small ones are outlined by yellow rectangles. Thereinto, the numbers of pixels occupied by the small ships are less than 20.

### 3.2. Detection Performance and Comparison

To carry out qualitative and quantitative comparisons of ship detection, seven comparative methods were used (i.e., the PMS [10], the PNF [15], the Pt-Ps [12], the RS [11], the SPAN [9], the DBSPc [13], and the VC-CR [14]). To comprehensively assess the detection performance, the figure of merit (FoM) is applied [33]:

(15) $$FoM=\frac{N_{\mathrm{tt}}}{N_{\mathrm{tt}}+N_{\mathrm{fa}}+N_{\mathrm{mt}}}$$

where $N_{\mathrm{tt}}$, $N_{\mathrm{fa}}$, and $N_{\mathrm{mt}}$ denote the numbers of correctly detected ships, false alarms, and missed ships, respectively. Here, $N_{\mathrm{gt}}=N_{\mathrm{tt}}+N_{\mathrm{mt}}$ is the actual number of ships provided by ground truth.

**Figure 3:**
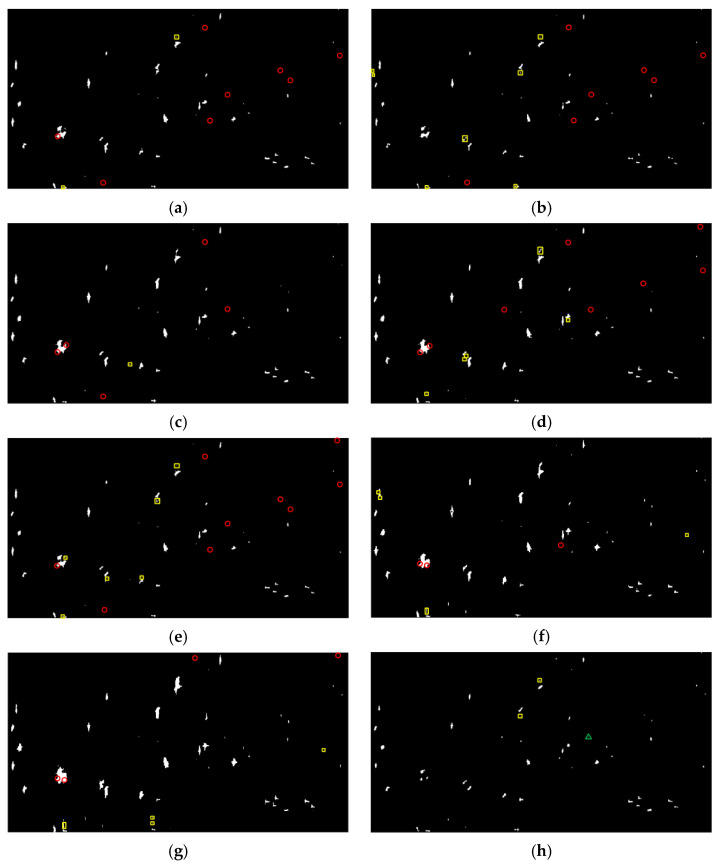
Ship detection results on GF3 data. (**a**–**h**) The PMS, PNF, Pt-Ps, RS, SPAN, DBSPc, VC-CR, and the proposed method, respectively. The red circles, yellow rectangles, and green triangles outline the missed targets, false alarms, and barely visible detected ships, respectively.

**Figure 4:**
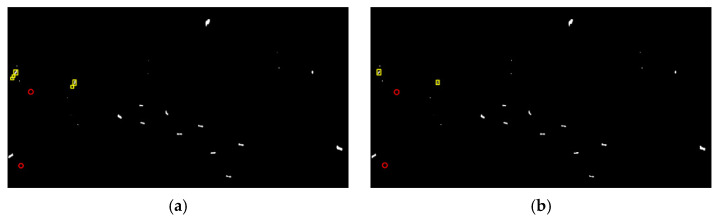

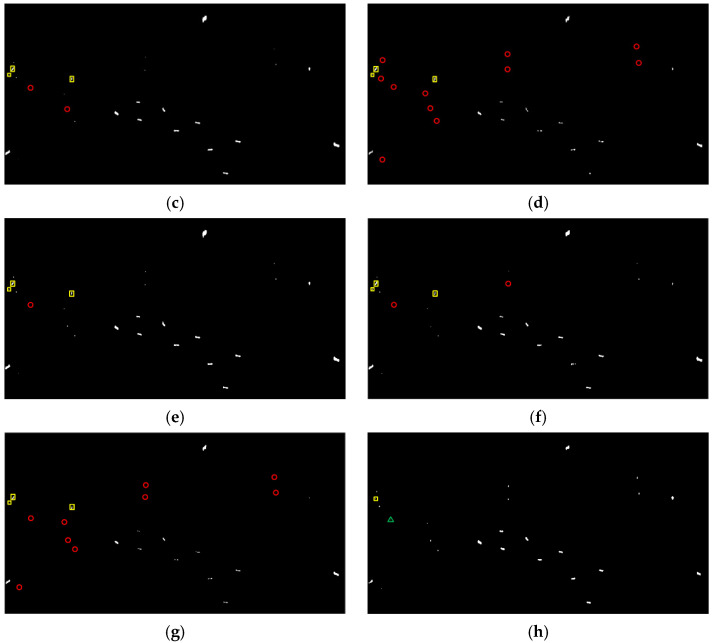
Ship detection results on AIRSAR data. (**a**–**h**) The PMS, PNF, Pt-Ps, RS, SPAN, DBSPc, VC-CR, and the proposed method, respectively. The red circles, yellow rectangles, and green triangles outline the missed targets, false alarms, and barely visible detected ships, respectively.

Generally, a higher FoM indicates a better detection performance. Figure 3 and Figure 4 present the detection results of different methods, where the yellow rectangle outlines the false alarms and the red circle outlines the missed ships. In addition, the barely visible detected ships are marked by the green triangle. It should be noted that the results are finally presented with a slight tuning of threshold according to the comprehensive consideration of the tradeoff between $N_{\mathrm{fa}}$ and $N_{\mathrm{mt}}$, which makes the FoM be the highest. The quantitative evaluation is summarized in Table 1. Notice that the statistical results presented here are the optimal results acquired through balancing the tradeoff between the detection probability and false alarms. Overall, all these methods can correctly detect large ships due to the strong backscattering. Meanwhile, false alarms also exist in these methods. They are mainly generated from sea clutter backscattering caused by surface wind or other oceanographic phenomena and are very difficult to remove. Despite this, the proposed method always produces the fewest false alarms (2 for GF3 data and 1 for AIRSAR data). This is attributed to the constructed ship detector excluding the contributions of surface and volume scattering, which are exactly the dominant scattering mechanisms of calm sea clutter and rough sea clutter.

What we can see is that there exist severe circumstances of missed ships for the comparative methods. The reason for the appearance of missed ships in the detection results is twofold. The first is that, after the thresholding, the adhesion of ship pixels occurs due to the existence of cross sidelobe, especially for GF3 data. The second and more is that some small ships only occupy a few pixels in the data, resulting in very poor visibility and detectability. This can be significantly observed in the RS method, which has lost all of the eleven weak ships in AIRSAR detection results. In contrast, it is impressive to find that the proposed method has no missed ships. In other words, the proposed method correctly detects all the true ships, indicating its robustness and effectiveness.

Taking the numbers of missed ships and false alarms together, the proposed method can achieve the optimal detection performance with a FoM of 0.96 for both of GF3 and AIRSAR data. Meanwhile, the FoM of other comparative methods are all less than 0.90, which demonstrates its superiority in small ship detection.

**Figure 5:**
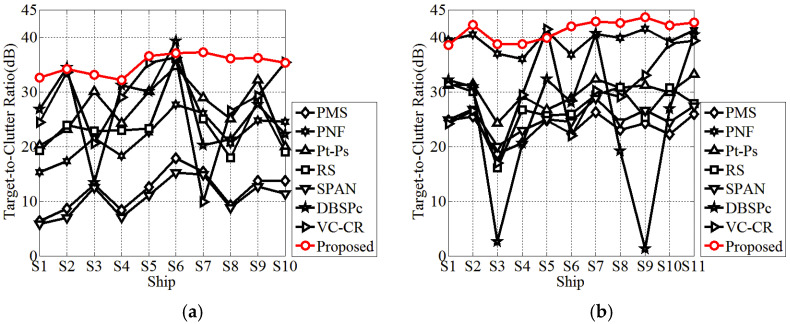
The target-to-clutter ratios for different ship detectors. (**a**) GF3 data. (**b**) AIRSAR data.

In the following, the TCR index is further applied for the quantitative evaluation of the highlighting of small ships. The TCR is defined as the ratio of detection feature averages between the ship and sea clutter (outlined by the white rectangles in Figure 2). All the TCRs are recalculated in decibels by 10 × log10 (TCR). The corresponding TCR distributions on small ships are given in Figure 5. It is obvious that the broken line of the proposed method is significantly above those of other methods, except for S1 and S5 in AIRSAR data, and S6 in GF3 data. As a matter of fact, for the comparative methods, the changes of TCRs are dramatic with respect to small ships (not only between different data but also among different ships in the same data). For instance, there exist sharp attenuations for the TCRs of S3 and S9 in AIRSAR data with respect to the DBSPc method, which leads to the missed detection (refer to Figure 4f). In contrast, the proposed method is more robust because, although the scattering powers of some small ships are weak, the weight difference between the local structure scattering and sea clutter scattering (surface scattering) is rather significant.

In statistics, the averaged TCR values for GF3 and AIRSAR data are 35.12 dB and 41.35 dB, respectively. Moreover, the biggest enhancement can even reach 41.46 dB (S9 in AIRSAR data compared with the DBSPc). This is attributed to the constructed ship detector adequately considering the scattering mechanism difference between the ship and sea clutter with the further amplification of the guard filter. What is noteworthy is that, for S1 in GF3 data and S3 in AIRSAR data (outlined by the green triangles in Figure 2 and Figure 3), the TCR differences are the most remarkable relative to all other methods, which explains why these two ships are both miss-detected for these methods. In summary, the proposed detector can enhance the TCR effectively, which benefits ship detection.

## 4. Discussion

### 4.1. Performance of the Proposed Decomposition

**Figure 6:**
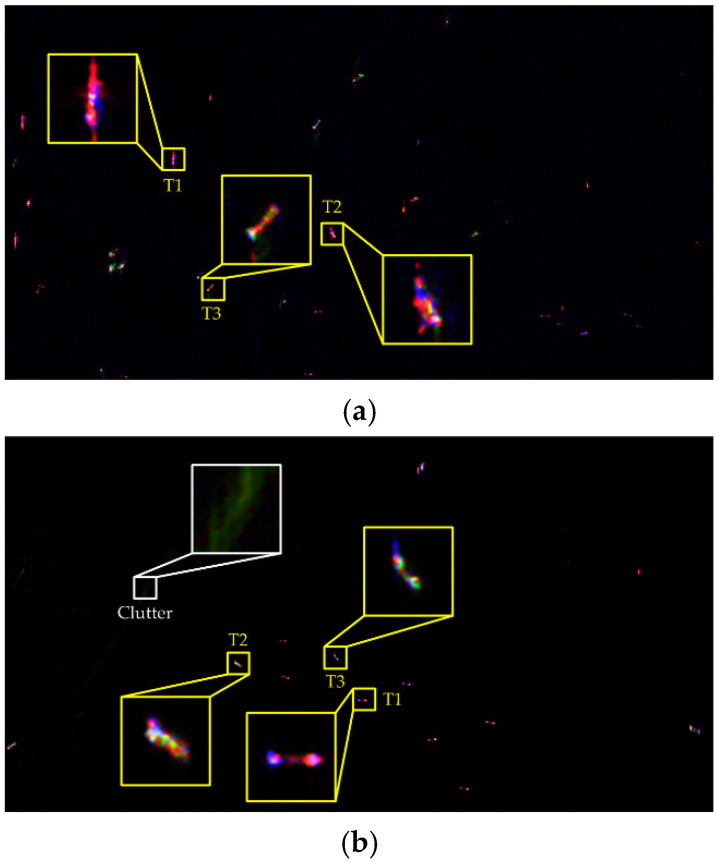
Fine eight-component decomposition results with several enlarged details (red: ship scattering, green: volume scattering, blue: surface scattering). (**a**) GF3 data. (**b**) AIRSAR data.

Next, the interpretation performance of the fine eight-component decomposition will be discussed. The color composite results for GF3 and AIRSAR data are given in Figure 6, where the red channel represents the ship scattering (the summation of double-bounce, helix, cross, ±45° OD, ±45° OQW, and MD scattering), the green channel represents volume scattering and blue channel represents surface scattering contributions.

As can be seen, ships exhibit various color distributions, indicating that ships with different orientations and structures have different scattering mechanism contributions. Meanwhile, the great amount of sea clutter is marked black and blue, which explains that the corresponding backscattering power is very low. However, for the rough sea clutter, multiple scattering interactions occur and obvious green tones can be observed (outlined by the white rectangle). It is noteworthy that the cross sidelobes and azimuth ambiguities are effectively suppressed, demonstrating the effectiveness of the proposed decomposition in terms of scattering mechanism discrimination.

To give a quantitative analysis, the statistics of normalized scattering contribution are implemented and presented in Table 2. To save space, three ships are respectively selected from GF3 and AIRSAR data since there are numerous ships. The corresponding decomposition details are zoomed in Figure 6. On a preliminary inspection, the proportion of double-bounce scattering is remarkably higher than others, demonstrating that most of the ships are colored red. This agrees with the reality since ships are mainly composed of dihedral structures, as hereinbefore introduced.

**Figure 7:**
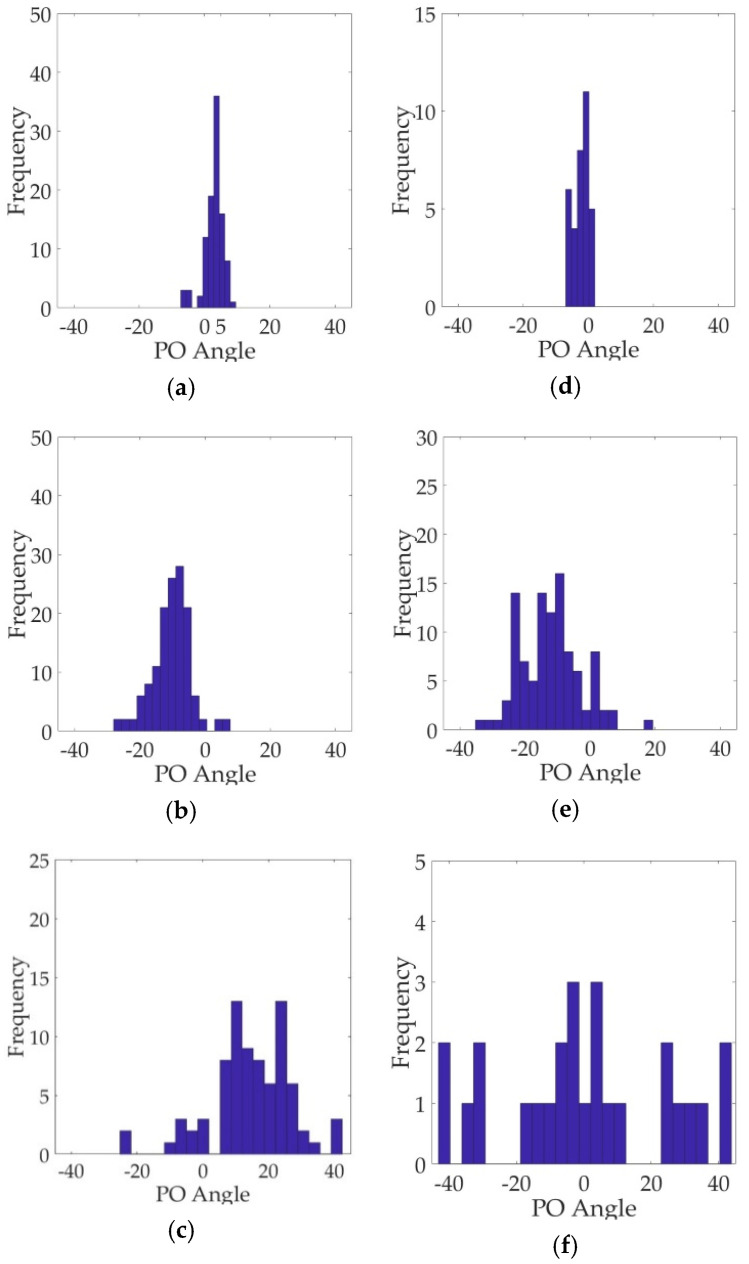
Polarization orientation (PO) angle histograms of ships. (**a**–**c**) Ships T1–T3 in GF3 data, respectively. (**d**–**f**) Ships T1–T3 in AIRSAR data, respectively.

For the purpose of comprehensively understanding the scattering mechanisms of ships, the histogram distributions of the PO angle of ships are further combined to discuss. As can be seen in Figure 7, for ships T1 and T2 in GF3 and AIRSAR data, the PO angle histograms gather closely and have distinct unimodal peaks. To be specific, the corresponding dominant PO angles are approximately 4°/−7° for GF3 data and −3°/−9° for AIRSAR data. In this case, the orientation shifts of ships are small and the co-polarization power is intense, which presents an obvious and dominant double-bounce scattering mechanism. Nevertheless, as the orientation shift increases, the proportion of double-bounce scattering decreases rapidly (about 25% and 36% for GF3 and AIRSAR data, respectively) and the volume scattering significantly enhances correspondingly. This accords with the fact that small variations in orientation will lead to abrupt change between co- and cross-polarization power, proving that the proposed method can considerably conform to the physical reality. Meanwhile, for ship T3, the range of PO angle distribution is wider and there exists multiple peaks in the histogram. In contrast with ships T1 and T2, the orientation shift is more apparent and the reflection symmetry of ship scattering is broken. At this point, the double-bounce scattering further decreases the local structure scattering, which is related to reflection asymmetry, increases instead. This can be interpreted as the scattering behaviors generated from the towers, guardrails, antennas and other dipole-like structures. In addition, the cross scattering power also increases, whose proportions are 4.93% and 3.08% for GF3 and AIRSAR data, respectively. This explains that considerable rotated dihedral scattering responses are produced due to the orientation shift.

Also notice that although the PO angles have exceeded the critical angle 22.5° [34] which invalidates the PO angle compensation for the MBD, the volume scattering power estimated from the proposed decomposition stays at a relatively low level and is below the ship scattering.

The above observation and analysis verify that the fine eight-component decomposition can not only accurately describe and present the scattering of local and complex structures of ships but also can effectively reduce the volume scattering and improve erroneous scattering mechanism interpretation. Accordingly, the constructed scattering contribution-based feature can well serve ship detection.

### 4.2. Window Parameter Test

**Figure 8:**
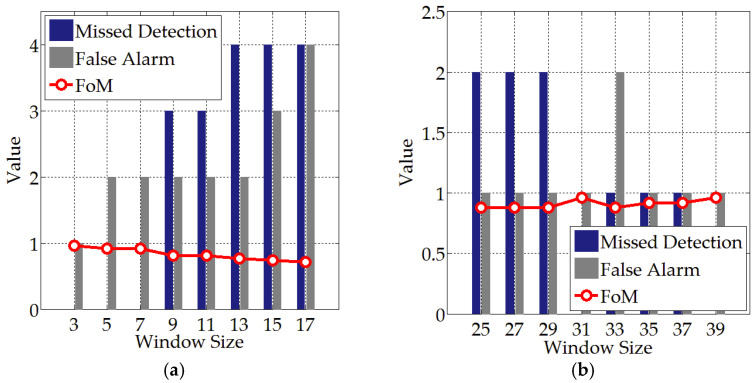
The impact on detection performance under different window sizes. (**a**) Size change of test window with a fixed guard window size (31 × 31 pixels). (**b**) Size change of guard window with a fixed test window size (3 × 3 pixels).

Although there are three parameters involved in the guard filter, the size difference between the guard and training windows is set to be a constant. Thus, only the detection results with different test and guard window sizes are compared. Taking the AIRSAR data as an example, and given a fixed size of 31 × 31 pixels for the guard window, the impact of the size change of the test window on the detection performance is investigated, as given in Figure 8a. It can be seen that the missed detection and false alarms increase with the increment of window size, resulting in the decrement of the FoM. This is because the increment of window size will bring in sea clutter pixels with respect to small ships, thus degenerating the performance of the proposed detector due to the influence of scattering contributions.

Similarly, to investigate the impact of size change of the guard window on detection performance, the test window size is fixed (3 × 3 pixels) and the comparison results are given in Figure 8b. It can be observed that when the guard window size is less than 31 × 31 pixels, there exist relatively more missed detection and false alarms. The detection performance reaches its optimal when the guard window size is 31 × 31 pixels. This is because according to the ship size and data resolution, the combination of the test window with a size of 3 × 3 pixels and the guard window with a size of 31 × 31 pixels guarantees that the guard filter can properly prevent the ship pixels from the contamination of sea clutters. Notice that, with the increment of guard window size, the FoM curve decreases and then increases to the optimal (39 × 39 pixels). However, a larger window size leads to a longer computation time. Accordingly, the sizes of the test window and the guard window are set as 3 × 3 pixels and 31 × 31 pixels, respectively in this work.

## 5. Conclusions

Using polarimetric techniques to detect ships for SAR images is of great interest in the field of military and civilian maritime surveillance. Given this, this paper designs a discriminative ship detector on the basis of fine model-based decomposition. On the one hand, four advanced scattering models correspond to rotated dihedral, ±45° OD, ±45° OQW, and MD structures are incorporated and a fine eight-component decomposition is put forward, which dedicates to depict the dominant and local structure scattering of ships. On the other hand, considering the scattering difference between the sea clutter and ships, the scattering contributions are combined to construct an identifiable feature and its efficacy is further amplified by a spatial information-based guard filter. Objective and subjective results on real PolSAR data demonstrate that the proposed method not only enhances the TCR of small ships and reaches the highest FoM of 0.96, but also validates and encourages the application of fine eight-component decomposition.

## Figures and Tables

**Table 1:** Quantitative Evaluations of Different Methods.

Data	Method	$N_{\mathrm{gt}}$	$N_{\mathrm{tt}}$	$N_{\mathrm{mt}}$	$N_{\mathrm{fa}}$	FoM
GF3	PMS	49	41	8	2	0.80
	PNF		42	7	7	0.75
	Pt-Ps		44	5	1	0.88
	RS		41	8	5	0.76
	SPAN		40	9	6	0.73
	DBSPc		46	3	4	0.87
	VC-CR		45	4	4	0.85
	Proposed		49	0	2	0.96
AIRSAR	PMS	24	22	2	5	0.76
	PNF		22	2	2	0.85
	Pt-Ps		21	3	2	0.81
	RS		13	11	3	0.48
	SPAN		23	1	3	0.88
	DBSPc		22	2	3	0.81
	VC-CR		15	9	3	0.56
	Proposed		24	0	1	0.96

**Table 2:** Scattering Contribution Statistics for Ships.

	GF3 Data			AIRSAR Data		
	T1	T2	T3	T1	T2	T3
Surface scattering	17.52%	28.13%	12.09%	28.37%	23.41%	27.84%
Double-bounce scattering	78.67%	53.34%	33.74%	62.00%	26.20%	11.50%
Volume scattering	2.98%	12.15%	28.65%	8.49%	28.49%	34.43%
Helix scattering	0.74%	4.60%	9.71%	0.93%	12.75%	10.24%
Cross scattering	0.03%	0.37%	4.93%	0.00%	1.86%	3.08%
±45° OD scattering	0.04%	0.87%	4.07%	0.00%	3.34%	7.32%
±45° OQW scattering	0.02%	0.48%	4.61%	0.21%	3.69%	5.59%
MD scattering	0.00%	0.06%	2.19%	0.00%	0.26%	0.00%
Ship scattering	79.50%	59.73%	59.25%	63.14%	48.10%	37.73%

## Data Availability

Data sharing not applicable.
